# The Expression Regulation Scale (ERS): Validation of Three Emotion Domains for Expressive Norms with Close and Distant Others in Private and Public Situations

**DOI:** 10.1177/10731911251333664

**Published:** 2025-05-06

**Authors:** Conal Monaghan, Yiyun Shou, Paige Mewton, Anika Quayle, Amy Dawel

**Affiliations:** 1 The Australian National University, Canberra, ACT, Australia; 2 Lloyd’s Register Foundation Institute for the Public Understanding of Risk, National University of Singapore, Singapore; 3 Saw Swee Hock School of Public Health, National University of Singapore and National University Health System, Singapore

**Keywords:** emotion, display rules, ant colony optimization, context, factor analysis

## Abstract

The social norms that guide emotional expression are critical for successful interpersonal interaction. However, the intricate emotional architecture underpinning these norms has remained largely unexplored. Our study is the first to rigorously investigate “display rules” or expressive norms for a comprehensive set of 64 theory-based emotions, utilizing a representative sample from the United Kingdom. The sample reflected national census demographics based on age, sex, and ethnicity. We measured expressive norms ranging from suppression to amplification in four social situations, combining two settings (public vs. private) and interactant types (close relations vs. distant others). Using a theory-building subsample (*n* = 507), we employed ant colony optimization (AOC) algorithms and a suite of factor analytical techniques to distill the emotions into three domains: affiliative, vulnerable, and disruptive. Subsequent validation in a separate confirmatory subsample (*n* = 506) supported this structure in all four situations (conditions), providing evidence these domains were robust. Notably, this new Expression Regulation Scale (ERS) demonstrated scalar invariance across all situations using repeated measures confirmatory factor analysis. We introduce scoring metrics and norms to aid researchers and practitioners in their analytical endeavors and highlight potential avenues for future research aimed at enriching our understanding of expression regulation.

## Public Significance Statement

A vital ingredient for successful social interaction is understanding and following the learned “display rules” that govern emotional expression. This study establishes a new scale for measuring these rules—the Expression Regulation Scale (ERS)—which can be used to understand their role in social relationships and well-being in different settings, including at work, home, and school, and in multicultural interactions.

Successful social interaction requires that people modify or regulate their behavior, including any outward expressions of emotion, to fit the normative expectations of the social situation at hand. For example, it may be socially inappropriate to express feelings of sadness by crying in a professional context, and doing so may have negative consequences, such as limiting career advancement. Yet, in an intimate situation with a close friend, this same emotional display might encourage bonding and support. The critical importance of these social norms for expression, also known as “display rules,” is highlighted by their prominence across psychological (Manokara et al., 2023; Matsumoto et al., 2008), anthropological (Lutz & White, 1986), and economics (Wang et al., 2011) research. Display rules also influence the potential for psychological burnout and other well-being outcomes (Gross & John, 2003; Wang et al., 2011). However, each of these fields has adopted idiosyncratic methods, which makes it difficult to synthesize the evidence base into a coherent narrative. The aim of the present study is to develop a psychometrically robust tool that can address this need by flexibly measuring expressive norms across different social situations—the Expression Regulation Scale (ERS; see Supplemental Material A for final instrument).

One reason for this methodological divergence is that, until recently, the emotional structure of expressive norms was largely untested. Although the specifics are debated, researchers minimally agree that emotions encompass both experiential components (e.g., heart racing, increased energy) and expressive components (e.g., smiling, frowning, pouting; Ekman & Cordaro, 2011; Matsumoto et al., 2005). Expressive displays can communicate underlying social intent, which may vary in the degree to which they align with the emotional experience but nevertheless carry social significance (Crivelli, & Fridlund, 2018). The nature of this social significance can be synthesized into emotional domains that capture the covariation between similar emotions and their associated social communication function.

In a study investigating public emotional display rules among Australian adults (*N* > 1,000), Dawel, Ashhurst, et al. (2023) used factor analysis of 24 theoretically grounded emotions to identify three distinct domains that shape expressive norms, each reflecting the social purposes these emotions serve: (1) fostering social harmony and affiliation (e.g., sympathy, joy); (2) signaling vulnerability and requesting support (e.g., sadness, fear); and (3) regulating social power/status dynamics, with potential to cause social disruption when expressed (e.g., anger, contempt). This initial structural work provides a foundation through which to build a robust understanding of display rules.

As expressive displays carry social significance, they must be understood within specific contexts. Prior research has consistently identified two key factors that influence expressive norms: the physical setting (public vs. private) and the relationship with the interactant (close vs. distant) (Manokara et al., 2023; Matsumoto et al., 2008). For example, expressing joy—an affiliative emotion—may strengthen bonds between close friends in private, while in public or with distant acquaintances, the same emotion might need to be expressed more subtly. These contextual factors can affect both the intensity and appropriateness of emotional displays, highlighting the importance of measuring expressive norms across different situations. Each social situation can also be seen within a broader context, which can include a range of factors such as the motivations and needs of the person. A second aim of the present study was to provide a more extensive and rigorous test of this structure by comprehensively sampling emotion terms and evaluating the equivalence and stability of expressive norms across four key situations.

Comprehensively sampling emotion terms was critical for ensuring that our scale development captured the full range of emotional domains. This approach also ensured strong coverage of the full breadth of emotions within each domain, with an adequate number of emotions for each (see Sutherland et al., 2013, for the importance of construct coverage). Our comprehensive approach advances understanding beyond existing scales for measuring expressive norms. For example, the Display Rules Assessment Inventory (DRAI; Matsumoto et al., 2005) is constrained to Ekman’s seven basic emotions (Ekman & Cordaro, 2011), whereas our method allows for the exploration of a broader emotional spectrum. This enabled us to uncover the underlying domain architecture of these emotions, a capability not afforded by measures that focused only on these basic emotions.

Although Dawel, Ashhurst, et al. (2023) went some way to address this limitation, five or fewer emotions loaded on the vulnerable and disruptive domain, compared to 11 for the affiliative domain. As such, the number of items limited content coverage. To address this limitation, we sourced the literature for influential lists of emotions from different literatures, which we then compiled and refined to obtain broad coverage of the most common emotion terms (Supplemental Material B), ultimately testing expressive norms for 64 different emotions. Critically, the terms included a range of social (as well as basic) emotions, such as admiration, embarrassment, guilt, and jealousy.

Understanding whether expressive norms and their underlying emotion domains (affiliative, vulnerable, and disruptive) are perceived similarly across different social situations is crucial for the validity of our research. Constructs can have different meanings in different contexts, even if they share the same basic structure (Bornstein, 1995). For example, affiliative emotions might represent openness and intimacy in private settings with close others but might reflect politeness or social etiquette in public settings. Similarly, expressing fear—a vulnerable emotion—might signal a need for support in private but serve as a warning or call for caution in public. Therefore, situational factors might shape display rules beyond just differences in how much emotions are expressed; they might fundamentally change what these emotion domains mean in different situations.

To ensure that our three-factor model accurately captures expressive norms across different situations, we need to establish measurement invariance. Measurement invariance tests whether the relationship between specific emotions and their underlying domains remains consistent across different situations (Meuleman et al., 2023; Putnick & Bornstein, 2016; Steenkamp & Baumgartner, 1998). In other words, it helps us provide evidence that the emotion domains mean the same thing in private and public settings and with close and distant others. By testing measurement invariance across all four situations, we can provide evidence that the relationships between specific emotions and their respective domains remain stable, ensuring that any differences we observe are due to actual differences in expressive norms rather than changes in how the domains function.

Existing measures of emotion expression norms, such as the DRAI (Matsumoto et al., 2005), the Flexible Regulation of Emotional Expression Scale (FREE; Burton & Bonanno, 2016), and the Emotion Regulation Questionnaire (ERQ; Gross & John, 2003), have provided valuable insights into understanding display rules. However, these measures have several limitations that the ERS was designed to address, thereby advancing the literature in several important ways.

The DRAI assesses seven basic emotions using nominal responses that are later converted to scalar scores (Matsumoto et al., 2005). Scalar scores derived from these nominal responses often lack intuitive interpretability, as they do not conform to a clear metric of display intensity. For example, the DRAI’s six ordinal response options typically load on a single domain and are converted to a single set of scaled scores for analysis. One limitation inherent in this approach is the challenge of intuitively interpreting scalar scores, as they do not conform to a clear metric of display intensity (e.g., amplify = 1.10, express as felt = 0.94). The present study aimed to address these issues by using a rating scale that produces scores that measure the degree of amplification, as well as suppression, and that can be understood intuitively. Therefore, ERS scores can be directly interpreted to show how participants modulated their emotions in specific situations, providing clear and meaningful insights (e.g., participants slightly amplified their emotions in situation X, but more so in situation Y).

Alternatively, the FREE scale uses highly specific scenarios, which may not be relatable for all participants or applications. The FREE’s specificity to certain contexts limits its adaptability to other social situations. Furthermore, this scale focuses on perceived ability rather than explicit rules, using items that reference positive and negative affect rather than measuring specific emotions (Burton & Bonanno, 2016). On the other hand, the ERQ, widely used for measuring expressive suppression, does not measure amplification at all and combines all types of emotions into a single score without specifying the context (Gross & John, 2003). Furthermore, methods in the emotional labor literature are highly variable (e.g., Allen et al., 2014; Lin et al., 2021), warranting a unified measurement instrument that can encompass the needs of all literatures interested in this construct. These limitations of current tools underscore the need for a more comprehensive and adaptable measure.

Our newly developed ERS addresses these gaps by offering a robust, situation-sensitive tool that captures both suppression and amplification across a wide range of emotions and social situations. The ERS employs clear labeling at different levels of expression, such as “I keep my emotions to myself” and “When I am feeling positive emotions, I am careful not to express them,” providing specific reference points that enhance accuracy and reliability. Additionally, it measures a bipolar underlying construct for each item, enabling nuanced assessments of emotional regulation. This design facilitates the measurement of subtle differences in emotional suppression and amplification across various settings and relational dynamics.

The present study also addressed a third important limitation of previous research; that some have focused only on control or suppression of expressions (e.g., Dawel, Ashhurst, et al., 2023; Gross & John, 2003) missing the potential for over-expressing or amplifying expressions. For example, a display rule might require one to amplify a smile to politely greet someone they feel neutral about seeing. The DRAI addresses this limitation by providing six nominal response options: “show more than you feel it”; “express it as you feel it”; “show less than you feel it”; “show it but with another expression”; “hide your feelings by showing nothing”; “hide your feelings by showing something else.” However, it includes only a single option for amplification, “show more than you feel it,” which does not allow for measuring differences in the degree of amplification across emotions or situations. Capturing nuances in the degree of over-amplification is crucial for understanding emotional expression in daily life.

The present study aimed to address these issues by using a rating scale that produces scores that measure the degree of amplification, as well as suppression, and that can be understood intuitively. Specifically, participants rate how much they think they should express each emotion from *Express no emotion/hide my emotion completely* (−100) through *Express it as I feel it* (0) to *Express much more than I feel (100)*. Negative scores indicate the degree of suppression, positive scores the degree of amplification, and scores around zero indicate little to no expression modification or regulation.

## The Current Study

The primary purpose of this study was to identify a model of expressive norms (also known as “display rules”) and an associated measurement scale that could capture emotions comprehensively yet parsimoniously and provide meaningful information across situations. We began with two foundational aspects of social situations: setting (private, public) and relational closeness (close or distant others), as they are factors well-established to influence social interactions. This framework provides a robust basis for understanding expressive norms and sets the stage for future research to explore more nuanced factors such as cultural differences, social status, and emotional adaptability. Dawel, Ashhurst, et al. (2023) only measured one setting (public) and did not specify the type of interactant, therefore, this study also aimed to establish the first broader set of normative data, for UK residents, to guide future researchers.

The current study expanded previous research in three ways. First, by using a larger comprehensive range of emotions as measured by individual survey items, we were able to ensure our prior three-domain model (Dawel, Ashhurst, et al., 2023) had not missed additional domains because of under-sampling emotion space. Our comprehensive approach also allowed us to identify additional emotions for the vulnerable and disruptive domains, which were captured by five or fewer emotions in Dawel, Ashhurst, et al. (2023). Second, we included emotional amplification versus suppression, which enabled us to capture the bi-directional nature of expression modification and produce scores that can be intuitively understood (positive scores = amplification; negative scores = suppression). Third, we used contemporary psychometric approaches to identify a robust factor (domain) architecture that could generate meaningful cross-situational information through a CFA invariance framework (Steenkamp & Baumgartner, 1998).

To achieve this, we collected data from a large sample of English-speaking UK resident nationals (*N* = 1,013), that was a representative sample based on census data for age, sex, and ethnicity. Initially, we randomly split the sample in half to perform exploratory and confirmatory analyses. Once scalar measurement invariance was established between the exploratory and confirmatory subsamples, we rejoined the samples to produce the normative data. Combining the two subsamples allows for greater statistical power and robustness in subsequent analyses, thereby providing a more comprehensive basis for developing normative data. We compared estimates from our final ERS to the ERQ (Gross & John, 2003), which asks participants to self-report how much they habitually use expressive suppression (with no specificity for any particular emotion). Prior work has found small to moderate correlations between the ERQ suppression scale and expressive norms/display rules (e.g., for the DRAI; Matsumoto et al., 2005). This comparison acted as both validation and theory advancement using the new expressive regulation architecture.

## Method

### Transparency and Sample Size Requirements

We report how we determined our sample size, and data exclusions, manipulations, and measures in the study. We follow Journal Article Reporting Standards (Kazak, 2018). Data were analyzed using R (R Core Team, 2021). The dataset is available at https://osf.io/tb6aq. This study’s design and its analysis were not preregistered; however, all analyses performed on the data are reported without exception.

Our sample size was determined *a priori* based on the requirements of factor analysis. For Exploratory Factor Analysis (EFA), recommended sample sizes vary widely, but Monte Carlo simulations suggest that stable parameter estimates for most underlying structures can be estimated with 400–500 participants (Kyriazos, 2018). To ensure that any identified structures were not measurement artifacts, we randomly divided the data into theory-building (exploratory) and theory-confirming (confirmatory) subsamples. To meet data requirements, we aimed to collect data from 1,100 participants to obtain a minimum of 500 per subsample, having estimated that approximately 10% of participants’ data would be excluded (for failing attention check or >10% missing data on at least one of the expressive norms tasks).

For CFA, we identified the sample size to discriminate between root mean square error of approximation (RMSEA) estimates (effect size) of .08 (H1) and .05 (H0), with an alpha = .05 and a power of approximately 1. Our sample size estimates of 500 met this requirement for both a final model of 10 items across each of the final three factors (*df* = 402) and a reduced design of eight emotions per domain (*df* = 249; Jak et al., 2021; MacCallum et al., 1996).

### Participants

The sample of UK residents was recruited using the Prolific crowdsourcing platform (https://www.prolific.co/). Quota sampling was used to ensure the sample matched the distributions of the census data for age, sex, and ethnicity. Inclusion criteria also required participants to be born and raised in the UK and speak English as their first language or fluently. Data from an additional 91 participants were excluded because they failed more than one of the six attention checks (83 participants) or had >10% missing data on at least one of the display rule situations seven participants) or both (one participant). Participants were compensated £4.00 for the ∼30-min survey. The study was approved by the Australian National University Human Research Ethics Committee.

Table 1 describes the total analyzed sample and the exploratory and confirmatory subsamples, selected by random assignment. Table 1 also shows that the subsamples were well-matched on key demographic variables, with no significant differences.

**Table 1:** Sample Demographics.

Characteristic	Total sample	Exploratory	Confirmatory	*t/χ* ^2^	*p*
*N*	1,013	507	506		
Men *N* (%)^a^	497 (49.1%)	247 (48.7%)	250 (49.4%)	.018^b^	.893
Women *N* (%)	510 (50.3%)	256 (50.5%)	254 (50.2%)	.007^b^	.933
Age *M* (SD)	45.7 (15.8)	45.6 (15.9)	45.8 (15.7)	.277	.782
Age range	18–82	18–81	18–82		
White *N* (%)	896 (88.5%)	441 (87.0%)	455 (89.9%)	.219^b^	.640
English as first language *N* (%)	930 (91.8%)	459 (90.5%)	471 (93.1%)	.155^b^	.694

a The response options for gender were: male, female, prefer a different term, or prefer not to say. Men indicate participants who identified as male. Women indicate participants who identified as female.

b Indicates *χ*^2^ was used.

### Study Design

Expressive norm situations (conditions) combined two settings (private, public) and two interactant types (close relations, distant others). The four situations were presented in counterbalanced order across participants and the order of the 64 emotions was randomized within each situation for each participant. Participants completed all situation conditions (in counterbalanced order) first, then demographics (age, gender, ethnicity, country of residence, number of years lived outside the UK before 18 years of age, English language fluency, ethnicity, household income), and finally the validity scale (Emotional Regulation Questionnaire; Gross & John, 2003). This was followed by some other questionnaires that are beyond the scope of the present study (see for full study survey).

### Expressive Norm Rating Task

Participants rated how they thought they should express each of the 64 emotions in the four situations: (1) in private with people very close to them; (2) in private with people not so close to them; (3) in public with people very close to them; and (4) in public with people not so close to them. Questions were customized for each situation, and each emotion was operationalized as a single survey item. For example, the private/close condition comprised the following instruction: “For this task, we would like to know how you think you should express emotions in private (for example, in either your own home or someone else’s) with people very close to you (for example, family and close friends). Please take a moment, while the below clock counts down, to imagine interacting in private with people very close to you.” These instructions remained onscreen for a minimum of 15 s to encourage participants to imagine the setting and interactant.

For each emotion, we used a visual analog scale from −100 to 100, anchored by five qualitative labels: *Express no emotion/hide my emotion completely* (−100); *Express less than I feel* (−50); *Express it as I feel it* (0); *Express more than I feel* (50); and *Express much more than I feel* (100). The scale had a default value of 0; however, a score on any item would only be recorded if the slider bar was clicked by the participant. If participants left the item at 0 without interacting with it, it was recorded as missing (see Supplemental Material A for an example of this scale).

#### Selection of Emotions

We aimed to investigate expressive norms for a comprehensive range of emotion terms to extend our previous work using 24 emotions from Keltner et al. (2019; see Dawel, Ashhurst, et al., 2023). To select the emotions, we first compiled 22 influential emotion lists from the psychology and linguistics literature, including lists from cross-cultural research (Bänziger et al., 2012; Barrett et al., 2007; Bednarek, 2008; Church et al., 1998; Cowen & Keltner, 2020; Ekman & Cordaro, 2011; Fontaine et al., 2007; Fredrickson, 2013; Izard, 1977; Jackson et al., 2019; Keltner et al., 2019; Lee, 2010; Masuda et al., 2012; McNair et al., 1971; Russell, 1983; Scherer, 2005; Shaver et al., 1987; Shiota et al., 2014; Van Goozen & Frijda, 1993; Wierzbicka, 1992). We refined the compiled list by converting all terms to the tense required for our expressive norm rating task (e.g., converting “angry” or “angered” to “anger”), retaining only those terms that appeared in at least two original lists, and by removing broad terms (e.g., “good” or “bad”) and terms relating to physical states (e.g., “hungry”). This process produced a master list of 174 emotions, which we further refined to 64 by retaining only those terms that appeared in at least four of the original lists and opting for the more common of any synonyms (e.g., preferring “fear” over “fright”). (https://osf.io/tb6aq reports the full compilation of lists and final 64 emotions.) We further considered frequency (Norvig, 2009; Ogren & Sandhofer, 2021; Shaver et al., 1987; Van Goozen & Frijda, 1993), age of acquisition (Baron-Cohen et al., 2010), cross-cultural use (Dewaele & Pavlenko, 2002; Hupka et al., 1999; Kaneko et al., 2006), and valence and arousal rating data (Grühn, 2016) when selecting and evaluating the emotions. Critically, the final list of 64 emotions includes the full range of valence and arousal covered by the compiled list of 174 emotions, indicating that coverage was comprehensive.

### External Validity Measure

#### Emotion Regulation Questionnaire

We used the four items from the ERQ (Gross & John, 2003) that measure habitual use of expressive suppression: *“I keep my emotions to myself*,”*“When I am feeling positive emotions, I am careful not to express them,” “I control my emotions by not expressing them,”* and *“When I am feeling negative emotions, I make sure not to express them.”* Note that one item refers specifically to positive emotions and one to negative emotions, but total scores for this scale are not specific to any emotion. Participants responded using a 7-point Likert scale from *strongly disagree* (1) to *strongly agree* (7). Item scores were summed to produce a total score for expressive suppression, with higher scores indicating greater use of expressive suppression. Estimates of internal consistency were strong and equivalent in both subsamples (Cronbach’s α = .80).

### Analytic Strategy

#### Factor Identification

We aimed to identify a factor structure that was stable and invariant across situations, facilitating meaningful inferences to be drawn about their comparison. All model identification and modification occurred solely in the theory generation (exploratory) subsample. First, EFA was used to identify substantive factor structures separately within each situation, resulting in four independent analyses. Given Dawel, Ashhurst, et al. (2023) used a smaller set of items, EFA was necessary to determine on which domains each of the new emotions loaded. We used a combination of the Elbow Test (Cattell, 1966) and Parallel Analysis (2,000 replications, quantile = .05; Horn, 1965) to determine viable factor structures, given they appear relatively robust to over-dimensionalization (van der Eijk & Rose, 2015) and can be supplemented with non-graphical interpretations such as the acceleration factor (the second derivative of the curve) and optimal coordinates (comparison of sample and factor analysis eigenvalues; Raîche et al., 2013). Factor structures were estimated using the psych (Revelle, 2023) and nFactors (Raîche & Magis, 2022) packages for R.

We then extracted possible factor structures and evaluated their quality using patterns of factor loadings and communalities, corrected item-total statistics, and inter-factor correlations. Given that expressive norms were expected to covary, we used oblique (Oblimin) rotation methods and determined final factors by integrating viable factor solutions with theoretical models (e.g., Dawel, Ashhurst, et al., 2023).

#### Emotion Reduction

To increase the parsimony of the final scale, we reduced the 64 emotions to the strongest archetypes of each factor, selecting 6–10 items based on their performance, with a strong emphasis on maintaining the diversity of emotions within each domain. Given the large number of possible permutations, ranging from 5.32e^13^ to 2.98e^15^, manually testing factor models was unfeasible as it would overwhelm even computerized brute force methodology. Therefore, we employed ant colony optimization algorithms (ACO; Deneubourg et al., 1983; Dorigo et al., 1991; Dorigo & Stützle, 2010) to efficiently resolve complex parameter spaces and identify psychometrically robust item subsets (e.g., Kerber et al., 2022; Olaru & Danner, 2021; Schultze & Eid, 2018).

ACO provides a stochastic protocol that imitates the pheromone trail-laying behavior of particular ant species. To identify the optimal display rule measurement models, computerized ants start by searching random paths for food (item sets). Routes that identify more attractive food sources (better model fit estimates) lay pheromones that increase the likelihood that future ants will follow the same path (select the items in that set). To reduce the probability of converging on poorer solutions, pheromones evaporate over time, allowing better-performing items to be selected with increasing likelihood until an optimum solution is produced. By running multiple chains simultaneously, ant colony algorithms enhance the search process by exploring several paths at once, reducing the risk of getting stuck in suboptimal, locally best solutions and increasing the chance of finding the globally optimal solution.

Using ACO to identify measurement models that were invariant across all four situations had the additional benefit of not being biased by a particular situation. That is, we assigned emotions to their respective factor based on EFA loadings. Then, this model was constrained to varying levels of equivalence across each situation using a repeated measures protocol. Traditional approaches involve finding strong solutions in one situation, then seeing if this solution is invariant across the other situations. In this way, ACO can outperform alternative item reduction techniques (Olaru et al., 2015) because it identifies solutions across all situations simultaneously.

To ensure content breadth (Dawel, Ashhurst, et al., 2023; Keltner et al., 2019) and avoid over-fitting in the final solution, we employed a theory-driven heuristic—a problem-solving rule or strategy that provides a practical way to guide decisions—based on a set of 17 selected emotions. This heuristic permanently increased the base likelihood of selecting these emotions in the ant colony optimization algorithm, serving as a fixed, theory-driven preference. Specifically, the heuristic favored these emotions but was intentionally weak, meaning it had a low influence on the ants’ decisions, allowing for more exploration of the search space. Unlike pheromones, which adapt based on past performance, the heuristic provided a constant nudge toward theory-driven emotions while still allowing the algorithm to explore other items and avoid premature convergence on local optima (see Supplemental Material B for more details).

As we placed a strong emphasis on measurement invariance, ACO was specified to optimize solutions that were scalar invariant across all four situations within the repeated measures CFA framework based on RMSEA and comparative fit index (CFI). That is, identifying items that were scalar invariant on their respective factors. Establishing measurement invariance in CFA involves a sequence of steps, each introducing more stringent constraints on the structure across different situations. It’s imperative to establish each step fully before advancing to the next (an exception here is partial-invariance which was not a focus of this study). First, configural invariance demands the same pattern of loadings, the significant and non-significant loadings of the items on their allocated factor, across all situations. Next, metric invariance constrains the strength of item loadings of those factors between situations. If loadings are equivalent, it suggests that the emotions represent their allocated latent factors similarly between each situation. However, systematic biases in emotion endorsement between situations can still skew mean comparisons at the metric invariance level. To account for this issue, the scalar invariance test constrains intercepts to be equal across situations, thus identifying whether systematic biases might obscure true group differences. For our study, achieving scalar invariance was paramount as it allows for meaningful cross-situation comparisons and legitimizes the comparison of latent means. If any of the target parameters are equivalent between situations, then constraining them to equivalence should not worsen model fit substantially.

We used best practices to guide measurement invariance analyses (e.g., Putnick & Bornstein, 2016), and model fit indices for all CFA models were: CFI and non-normed fit index (NNFI) > .950, RMSEA, and standardized root mean square residual (SRMR) <.080 and <.060, respectively (Hu and Bentler, 1999). Despite inconsistencies in recommendations for how small changes in fit estimates need to be for practical invariance between steps, we primarily focused on the most broadly used threshold of ΔCFI < .01 (Chen, 2007; Cheung & Rensvold, 2002; Kim et al., 2017), while also interpreting all tests in respect to more conservative recommendations, ΔCFI < .002 (Meade et al., 2008). To complement these analyses, we then ran CFA invariance analyses for each situation across gender identities and age groups. All ant colony algorithms were run using the Stuart Algorithmic Rummaging Techniques Package (Schultze, 2023) in the R Statistical Software (R Core Team, 2023), implemented using RStudio/Posit (Posit Team, 2023).

#### Model Refinement and Replication

We then evaluated the final models for sources of model strain and misfit (Brown, 2015; Sellbom & Tellegen, 2019; Waldman et al., 2023). This involved the magnitude consistency factor loadings, modification indicates, differentiation (latent covariances) between factors, residual covariances, and associations between factors and external relevant variables. Given the risk that ACO might identify a narrow set of emotions per domain to maximize the target modification indices, we aimed to use these sources of model misfit to identify redundant emotions and ensure that the content breadth was retained. After any modifications were made in the exploratory dataset, we estimated model fit and invariance in the Confirmatory dataset without modification using lavaan (version 0.6-18; Rosseel, 2012) and semTools packages (version 0.5-6; Jorgensen et al., 2008). Fit estimates in the Confirmatory dataset were used as a strict test of our model’s robustness and thus theory.

## Results

### Model Development

#### Exploratory Factor Analysis

Data exceeded guidelines for EFA appropriateness, at 7.91(cases):1(item), Kaiser–Meyer–Olkin estimates ranging from .96 to .97, and all Bartlett’s χ^2^ values were significant, *p* < .001. Factor identification based on both Scree and Parallel analysis (Supplemental Material C) suggested two strong factors explaining approximately 50%–60% of the variance in the data depending on the situation. The Scree test also indicated the possibility of a third factor accounting for an additional 5% variance. Parallel analysis indicated the possibility of up to four factors. Supporting these findings, the acceleration factor (the second derivative of the curve) suggested two factors, whereas the optimal coordinates (comparison of sample and factor analysis eigenvalues) suggested up to four. We therefore extracted two to four-factor solutions for further evaluation.

We rejected the four-factor solution as spurious because the fourth factor comprised only two to four emotions (loading <.32), depending on the situation, and contributed only an additional 2% of variance to each factor. It was likely a measurement artifact due to the number of initial emotions (van der Eijk & Rose, 2015). The two- and three-factor solutions had strong divisions of item loadings across all candidate factors in all situations, with a large proportion of these loadings exceeding .50. The pattern of factor loadings was similar across all situations, indicating the likelihood of an underlying set of invariant emotions. Inter-factor correlations were consistently higher in the Private-Close situation (see Supplemental Material D for EFA results). The factor correlations in the two-factor solution ranged from .23 to −.07. In the three-factor solution, the correlations between Factor 2 (capturing affiliative emotions) and the other two factors (capturing vulnerable and disruptive emotions) remained nonsignificant to weak (−.08 to .23), whereas the correlations between the factors capturing vulnerable and disruptive emotions were strong (.68 to .73).

### Item Reduction

We chose the three-factor solution because it included items that could be combined later to assess a two-factor structure by merging two of the factors, and because some key theoretical frameworks require a distinction between vulnerable and disruptive emotions for expressive norms (Crivelli & Fridlund, 2018; Dawel, Monaghan, et al., 2023; Flicker et al., 2019; Safdar et al., 2009). However, we include the results for the two-factor structure in Supplemental Material E for comprehensiveness.

We reduced the structure using ACO for the three-factor solution using theory-driven heuristics to guide the start of the model estimation. We ran one three-factor ACO, specifying each emotion from the EFA into their constituent factor, and specified the algorithm to identify 30 emotions (10 per domain) that minimized RMSEA, CFI, and ΔCFI (Supplemental Material B). We ran the ACO on the exploratory sample only, as this retained the confirmatory sample for later model validation and as a form of protection against over-fitting.

All emotions correlated strongly and consistently with their respective factor (> .70). However, modification indices suggested strongly correlated error covariances between several pairs of items. These emotion pairs were variations in intensity of the same underlying emotions, for example, stress and distress. In line with our analytical plan to identify a parsimonious model of expressive norms, we removed two items per domain that shared the same core emotion (correlated residuals) with more strongly loading items.

### Final Emotion Domain Models

**Figure 1.**
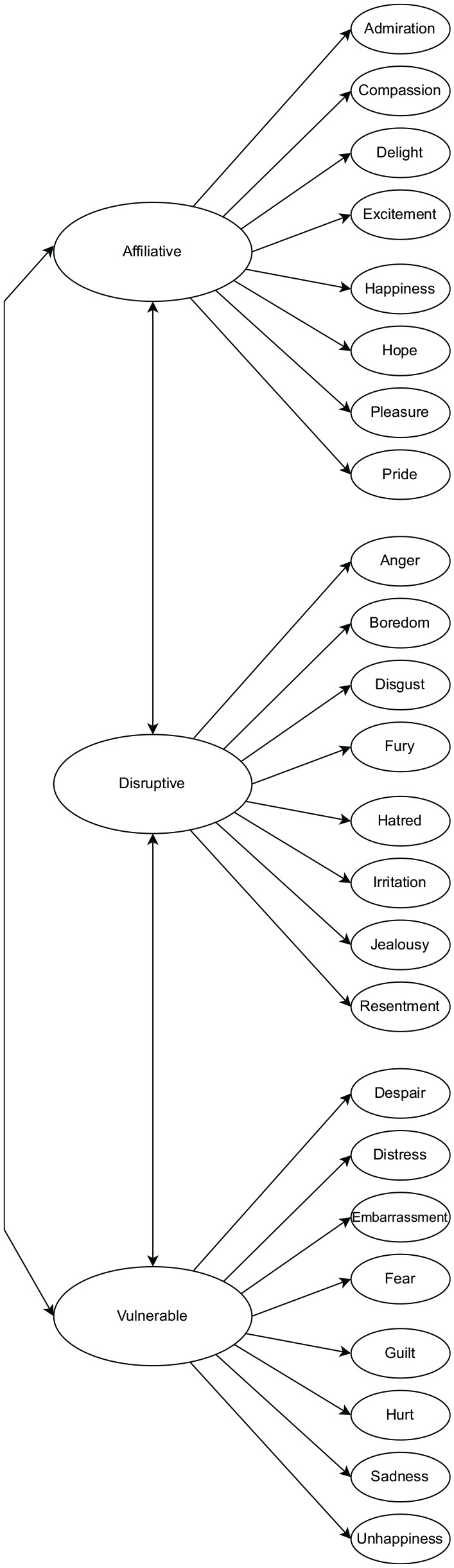
Conceptual Structural Model for the Final Expression Regulation Scale. *Note.* Oblique shapes used for indicators over rectangles to represent conceptual structure, not empirical structure.

We then investigated the final models which comprised of three sets of eight emotions in both the exploratory and confirmatory subsamples. The final factors closely resembled those identified by Dawel, Ashhurst, et al. (2023). However, we modified the domain labels to better reflect the expanded set of underlying emotions; now affiliative (originally harmonious), disruptive (disharmonious), and vulnerable. Estimates of internal consistency (ω_total_) were strong and consistent across the samples, ranging from .91 to .95 across situations (see Supplemental Material F). Confirmatory Factor Analyses using Robust Maximum Likelihood estimation were then run in each situation separately. The three-factor model had acceptable estimates of model fit indices across all situations (Table 2), which replicated in the confirmatory sample, suggesting the model is robust and replicable.^1^ The final structural model can be seen in Figure 1 (see Supplemental Material F for emotion loadings for each situation), and the final 24-item ERS in Supplemental Material A.

**Table 2.** Model Fit Indices from Confirmatory Factor Analytic Models.

Model	Model fit estimates						
Situation	χ^2^	*df*	*p*	RMSEA	SRMR	CFI	NNFI
Exploratory sample							
Private-close	866.71	249	<.001	.058	.053	.956	.952
Private-distant	590.15	249	<.001	.040	.058	.975	.972
Public-close	752.01	249	<.001	.054	.046	.959	.954
Public-distant	732.63	249	<.001	.054	.057	.956	.951
Confirmatory sample							
Private-close	841.77	249	<.001	.055	.056	.960	.955
Private-distant	692.66	249	<.001	.049	.054	.960	.956
Public-close	732.21	249	<.001	.053	.047	.956	.951
Public-distant	720.50	249	<.001	.052	.047	.955	.951

*Note*. Estimator = MLR.

We ran repeated measures CFA for the proposed three-factor structure (Table 3). In both the exploratory and confirmatory subsamples, the fit indices for configural, metric, and scalar models were within acceptable ranges. Although the ΔCFI exceeded the more stringent criteria (ΔCFI < .002), ranging from ∼.000 to .006, it complied with the commonly accepted threshold (ΔCFI < .01). This provides evidence that the mean comparisons across various situations were unbiased, both in terms of the interpretation of each domain (metric invariance) and their thresholds (scalar invariance).

**Table 3:** Fit Estimates from Repeated Measures Invariance Modeling for Three-Factor Emotion Domain Models.

		Model fit indices				
Dataset	Configural	χ^2^	df	RMSEA	CFI	ΔCFI
Exploratory						
	Configural	6544.54	4254	.037	.928	
	Metric	6627.36	4317	.037	.928	.000
	Scalar	6830.51	4380	.038	.924	.004
Confirmatory						
	Configural	6704.74	4254	.038	.919	
	Metric	6798.34	4317	.038	.918	.001
	Scalar	7005.87	4380	.039	.913	.005

*Note.* Estimator = MLR.

Finally, we extended the invariance analysis to include binary gender identity (due to insufficient sample size for non-binary identities) and age, combining the exploratory and confirmatory samples at this point. For each sequential level of restriction (configural, metric, and scalar), the three-factor ERS was invariant across binary gender identities for all four situations (ΔCFI = .000–.003). As our approach to invariance requires comparison groups, we divided age into three groups: <−1 SD (29 years, *n* = 216), −1 to 1 SD (30–61 years, *n* = 591), and >1 SD (>62 years, *n* = 206). The ERS was invariant across the three age groups for all four situations, for both the three-factor (ΔCFI = .000–.008) and two-factor (ΔCFI = .000–.007) models (see Supplemental Material G for detailed analysis output). These results further support the robustness of the ERS and demonstrate its capacity to provide meaningful comparisons across age and gender identities.

### Expressive Norms Across Situations

We used the combined dataset (exploratory and confirmatory) for validity tests. Across all situations, affiliative emotions were consistently endorsed as being over-expressed, with a positive skew toward amplification. Interestingly, the level of expression for affiliative emotions was consistent, with medians only ranging from 24.44 (close-private) to 9.56 (distant-public), and few participants endorsed any level of suppression.

In contrast, both vulnerable and disruptive emotions were more dispersed, and suppression of them was generally endorsed across all situations. This trend was pronounced in all situations except for close-private, which appeared more symmetric. The endorsement of suppression tended to be greater for disruptive than vulnerable emotions across all situations, though this difference was relatively minor (approximately 10 units in all situations). The most distinct contrast between vulnerable and disruptive emotions emerged in the distant-public situations. Here, disruptive emotions were positively skewed, with a mode nearing −100, suggesting a strong preference for suppression. Conversely, vulnerable emotions displayed a mode between −30 and −40, with a flatter distribution below 0, implying more varied beliefs about how these emotions should be expressed in distant-public situations.

**Figure 2.**
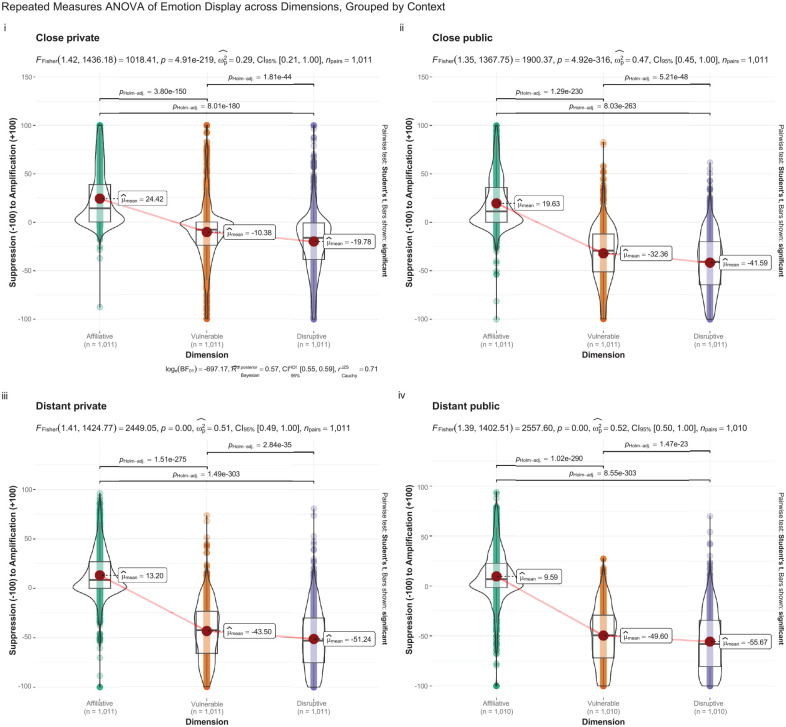
Distribution (Violin) Plots and Repeated Measures ANOVA of Expressive Norms by Emotion Domain, Grouped into Panels by Situation. *Note.* BF = Bayes Factor.

One-way repeated measures analysis of variance (ANOVA) suggested that, within each situation, there were significant differences in expressive norms between each emotion display rule domain (see Table 4; Figure 2). The effect sizes were moderate (*F*-statistics; see Figure 2) and the Bayes Factor for the same analysis suggested that the data were substantially more likely (>600 times) under the alternative hypothesis as compared to the null hypothesis. Post-hoc *t*-tests also suggested that all pairs of display rule domains differed significantly.

**Table 4:** Product–Moment Statistics for Display Rules Between Situations.

Domain	Affiliative				Vulnerable				Disruptive			
Situation	Median (IQR)	M (SD)	Skew	Kurtosis	Median (IQR)	M (SD)	Skew	Kurtosis	Median (IQR)	M (SD)	Skew	Kurtosis
Total	10 (0, 31)	17 (27)	0.51	1.55	−32 (−57, −10)	−34 (33)	0.16	0.33	−42 (−68, −18)	−42 (33)	0.29	0.02
Private-close	14 (0, 39)	24 (29)	0.95	0.13	−8 (−24, 0)	−10 (30)	2.21	0.94	−16 (−38, −1)	−20 (32)	1.31	0.99
Private-distant	11 (0, 36)	20 (26)	0.13	2.22	−30 (−52, −12)	−32 (29)	0.14	−0.17	−41 (−65, −20)	−42 (30)	0.42	0.07
Public-close	9 (0, 27)	13 (24)	0.75	0.88	−43 (−66, −24)	−43 (29)	−0.04	0.29	−53 (−75, −30)	−51 (30)	0.07	−0.41
Public-distant	7 (−2, 23)	10 (26)	−0.16	2.14	−49 (−72, −29)	−50 (28)	0.05	−0.77	−58 (−81, −34)	−56 (30)	0.45	−0.3
*p*-value	<0.001	<0.001			<0.001	<0.001			<0.001	<0.001		

*Note*. IQR = inter-quartile range. Median differences were estimated using Kruskal–Wallis rank sum tests; mean differences were estimated using repeated measures ANOVA. See Supplemental Material G for breakdown of estimates of central tendency into exploratory and confirmatory subsamples.

Given latent models across situations were reasonably scalar invariant, we extended these analyses through a latent modeling equivalent for a repeated measures ANOVA, an extension of latent growth curve modeling. This provides a range of additional benefits not afforded to manifest modeling, such as being able to explicitly model error and more complex covariance structures, in addition to allowing for model fit estimates and robust estimators (Langenberg et al., 2022; Mayer et al., 2012). We estimated models using the semnova package (v 0.1-6; Langenberg & Mayer, 2020). Given our aims to identify differences between each condition, we used effects-coding, fixing the scale through the weighted average of the indicators.

The model was a 2 × 2 factorial structure with interactant (close vs. distant) and setting (private vs. public) as within-subject factors (see Langenberg et al., [2022] for details on this approach regarding constraints and contrasts.) Across all three emotion display domains, significant main effects were found for both interactant and setting (*p* < .001). Therefore, both interactant and setting had significant influences on latent display rule domains. The interaction between interactant and setting was significant for the vulnerable and disruptive domains (*p* < .001), indicating that the effect of the interactant (close vs. distant) on emotions varied depending on whether the interaction occurred in a public or private setting. However, for the affiliative domain, the interaction effect was non-significant (*p* > .24), suggesting that affiliative emotions were influenced similarly by interactant and setting (Supplemental Material I for full ANOVA SEM results).

### External Associations

We then evaluated the relationship between the ERS domains and ERQ suppression (Table 5). Correlations with affiliative emotions were negative but weak across all situations. These associations were stronger for vulnerable and disruptive emotions, and stronger for private than public interactants.

**Table 5:** Correlations Between ERS and the ERQ Suppression Subscale.

Situation	Affiliative	Vulnerable	Disruptive
ERQ suppression			
Private-close	−.06	−.20***	−.16***
Private-distant	−.08*	−.19***	−.13***
Public-close	−.09**	−.12***	−.05
Public-distant	−.08**	−.14***	-.07*

*Note.* See Supplemental Material J for separate correlations for exploratory and confirmatory subsamples.

* *p* < .05. **p < .01. ***p < .001

## Discussion

The current study investigated the underlying emotional structure of the norms that guide emotional expression—otherwise known as display rules—in a large sample of the UK general public, matched to census data for age, sex, and ethnicity. Given the inherent interpersonal and contextual nature of display rules, we measured beliefs about how much an emotion should be expressed from suppression to amplification across four situations combining privacy and interactant closeness. We found that, across all situations, display rules can be conceptualized as organizing into three underlying emotion domains: affiliative, vulnerable, and disruptive, in line with our seminal work (Dawel, Ashhurst, et al., 2023). This modeling was used to establish the 24-item ERS (Supplemental Material A), with eight items capturing each of the three domains.

### Expression Regulation Scale

We followed contemporary approaches to robust psychometrics. This included randomly dividing the sample into theory-building (exploratory) and theory-testing (confirmatory) subsamples. Employing ACO, we efficiently narrowed down potential emotions while preserving a wide spectrum of content and diversity among the items. This was achieved through heuristic methods and content review. Subsequently, we demonstrated that the final model was scalar invariant, ensuring consistent measurement across various situations, genders, and age brackets. Notably, the application of ACO in our research proved superior to traditional manual item reduction techniques. Typically, manual methods are constrained by the researcher’s degrees of freedom and are less likely to pinpoint the strongest solution (Olaru et al., 2015). ACO’s effectiveness was particularly evident in identifying a stable factor structure across different situations—a task that poses significant challenges when using conventional methods, such as the systematic removal of constraints within a partial-invariance framework.

Although we endorse a three-domain model—due to better fit and for theoretical reasons (Dawel, Ashhurst, et al., 2023; Flicker et al., 2019; Safdar et al., 2009)—a two-domain solution was also viable (Supplemental Material E). This reflected the observed similarity in expressive norms between disruptive and vulnerable emotions, and the relatively strong correlation between these domains. Although disruptive and vulnerable expressive norms encapsulate unique forms of emotional communication with separate societal implications, they both capture negatively valenced emotions (Simpson & Stroh, 2004). Conflating these domains would, however, obscure their substantive differences, which were clearest in public settings or with distant interactants, and may be problematic for some theoretical frameworks (e.g., Crivelli & Fridlund, 2018; Dawel, Ashhurst, et al., 2023; Flicker et al., 2019; Safdar et al., 2009) for other situations where the difference may matter more (e.g., professional settings or cultures with larger power distances). These observations align with prior research that has underscored strong parallels between these two domains, yet also highlighted their divergence in specific situations.

For instance, Dawel, Monaghan, et al. (2023) discovered that although expressive norms for these two domains were similar in both online and face-to-face interactions with friends and co-workers, notable distinctions emerged when communicating with a supervisor, doctor, or psychologist, with this differentiation being more pronounced among British participants than Australians. There are also strong theoretical reasons to distinguish between vulnerable and disruptive emotions. An influential hypothesis concerning gender differences in emotion argues that norms allow for women to express vulnerability more than men, but for men to express dominant or disruptive emotions more (Dawel, Ashhurst, et al., 2023). A clear benefit of the ERS’s three-factor structure is that it enables such hypotheses to be tested, warranting the retention of three domains/subscales.

The use of a slider bar in our study offers several distinct advantages over traditional Likert-type scales. Our approach includes clear labeling at different levels of expression within the visual analog scale space, such as “I keep my emotions to myself” and “When I am feeling positive emotions, I am careful not to express them,” providing specific reference points that enhance the accuracy and reliability of responses (Furr, 2011). Additionally, the ERS measures a bipolar underlying construct for each item, enabling nuanced measurement within both amplification and suppression. For example, facilitating the measurement of subtle differences in emotional suppression between interactions with friends versus intimate partners. Simms et al. (2019) caution that visual analog scales may not offer psychometric advantages over traditional Likert-type items, highlighting the need for careful consideration and pilot testing of response formats. Our use of a visual analog approach was carefully matched with theory and the need for measurement precision over a wide range; however, the increased precision gained through our use of a visual analog over Likert-type scale is an empirical question that was not directly tested in this research.

Importantly, both the three-domain solution was scalar-level (and preceding levels) invariant across each of the four situations. This robust invariance across situations suggests that the underlying factor structure of the emotional domains remains consistent irrespective of the varying relational (interactant) and social settings. These findings provide a solid foundation for making meaningful comparisons of latent means across these situations. Previous work that compared interactant and setting (e.g., Manokara et al., 2023; Matsumoto et al., 2008) did not establish invariance and therefore it was unclear whether differences across situations were due to measurement bias or substantive effects. Invariance also implies that the cognitive appraisal or internal representations of these emotional domains might be universally consistent, regardless of external situational demands.

The internal consistency of each domain (ω_total_) across all situations and both samples exceeded .90. This suggests that the scale can provide precise estimates of expressive norms, suitable for investigating individual differences, as well as between-group designs. It is notable that this consistency was still achieved even though we prioritized breadth over internal consistency when selecting emotion items, ensuring the selected emotions represented the full spectrum of emotions. Also, given each subscale was only eight items long, this level of consistency is promising given these estimates tend to increase with scale length and decrease upon replication (Tavakol & Dennick, 2011). This tendency arises because, even if newly added items are of poor quality, they can still increase parameter stability. It might be tempting for researchers to reduce the scale given the pressure for shorter scales in psychological research (Rammstedt & Beierlein, 2014). However, our final choice of an eight-item-per-subscale approach offers comprehensive coverage and stable results that are harder to achieve with shorter scales, which are more influenced by individual responses to specific emotions.

### Display Rule Distributions

Although individual emotional displays may manifest at different intensities based on specific settings and the depth of interpersonal trust and familiarity, a baseline pattern of expressive norms was evident for the three foundational emotion domains. Expression norms for affiliative emotions ranged from express-as-is to amplification, whereas norms for vulnerable and disruptive emotions tended toward suppression across all situations, with very few participants believing these emotions should be over-expressed. The most notable difference between vulnerable and disruptive emotions occurred in public with distant interactants, where the tendency for suppression was notably stronger for disruptive than vulnerable emotions.

The patterns across situations were similarly striking. As previous research has found (Manokara et al., 2023; Matsumoto et al., 2008), there was a tendency toward greater expression in private than in public, and with close than distant others. However, it is not the case that people express all emotions most authentically in private settings with people close to them, as these settings produce greater levels of over-expressing affiliative emotions. This interaction highlights the importance of our research identifying different emotion domains for expressive norms as investigating this concept as a unitary construct would miss this nuance.

In a similar vein, the data suggested a distinct demarcation point along each display domain, characterized by a significant decline in display endorsement at certain junctures (illustrated in Figure 2). This trend was particularly notable in the context of affiliative emotions where there appeared a marked aversion to suppression, resulting in a discontinuity of endorsement at this point. A comparable trend was observed for disruptive and vulnerable emotions, where there was a notable scarcity of participants endorsing the amplification of these emotions. This pattern delineates a stark, almost categorical boundary within the behavioral norms that govern emotional expression, intimating the existence of widely held display rules that deem certain emotional expressions as inappropriate.

### Emotion Regulation

A notable outcome of this study was the identification of a weak to moderate correlation between ERS scores and ERQ suppression, which captures the habitual hiding of emotions. While these results are consistent with prior findings (e.g., Dawel, Ashhurst, et al., 2023), they suggest a discrepancy between perceived norms for emotional expression and the actual daily management of these emotions. Multiple factors might account for this discrepancy. One possibility is the divergence between how individuals handle their own emotions (as exemplified by the ERQ item: “*I keep my emotions to myself”)* versus an idealized perspective of personal or social behavioral expectations. Consistent with this notion, prior studies have suggested that individuals maintain distinct expressive norms for themselves in comparison to others when it comes to gender identity (Dawel, Ashhurst, et al., 2023). The infrequent experience of specific emotions might also play a role, because many ERQ items are conditional on experience, such as starting with “*When I am feeling…*”. Even if an individual seldom encounters a particular emotion (and thus rarely engages in suppressing it), they might possess robust beliefs about its appropriate expression. In contrast, the strength of the emotion likely dictates one’s capacity to purposefully choose how it is displayed, as people may have higher motivation to regulate the expression of more intense emotions, particularly negative ones.

### Limitations and Future Directions

Our findings reveal that expressive norms measured by the ERS show both consistency and variability across different social situations. While the ANOVA and latent ANOVA analyses indicated significant main effects for both setting (private vs. public) and interactant (close vs. distant), the correlations of the ERS domains across the four situations were high (see Supplemental Material H). This suggests that individuals may hold consistent expressive norms across various social situations, pointing to a generalizability in how people believe emotions should be expressed. However, the observed differences across situations, though sometimes subtle, can be meaningful depending on the research focus. For instance, expressive norms for vulnerable and disruptive emotions varied notably between private and public settings and between close and distant interactants. These nuances can have significant implications in areas such as cross-cultural research, organizational behavior, and interpersonal communication. For example, in organizational settings, employees may feel pressured to regulate emotions like anger or frustration more strictly in public or hierarchical interactions, while in private or among close colleagues, they may express these emotions more freely. This flexibility in adapting expressive norms to suit different social contexts could influence workplace dynamics, team cohesion, and conflict resolution strategies.

Although our findings are limited to four broad social situations, they suggest a potential universality of expressive norms that could guide future research to reveal the nuances of how norms vary across different expressers and contexts. Measuring emotional expressions in different situations can capture the variability and adaptability of individuals’ emotional display rules. For example, differences in display rules across cultures might be more pronounced in public than in private settings. Furthermore, assessing individuals’ flexibility in applying these rules can reveal important psychological insights, such as the potential for social difficulties when the same rules are used across all settings. Future research could explore additional factors that influence expressive norms, such as power dynamics, social implications, and peer pressure, to further understand the complexities of emotional expression in various social environments.

Our current situations did not vary the relative social status of the interactants, a factor that might induce greater differentiation between vulnerable and disruptive emotions given the differential implications of being disruptive or vulnerable with an employer or police officer. The ERS provides a psychometrically sound foundation for this future research, which should explore these domains to provide a more comprehensive understanding of emotional expression across diverse social contexts.

The present study applied the ERS to measure expressive norms (“display rules”) by asking participants how they thought they should express emotions in different situations. However, this wording could be adapted to target expressive behavior. For example, by asking participants how they actually express their emotions. The wording could also be adapted to capture habitual expression regulation, akin to that captured by the (ERQ; Gross & John, 2003); for example, by asking “When I feel the following emotions, I…”. However, the factor structure of the ERS is yet to be tested for these alternative wordings and there will be a need to reassess the validity of adapted versions of the scale.

Expressive norms are often driven by various goals, such as maintaining one’s appearance, nurturing relationships, or safeguarding personal agendas. In our current study, we did not explicitly account for these motivational factors, and it is likely that individuals’ motivations within different situations vary considerably. For instance, in close-private relationships, individuals may exhibit more relaxed and unfiltered expressions, while also making efforts to maintain positive social impressions to nurture these relationships. This intriguing possibility highlights the need to explore the role of individual differences in our research.

We modeled the effect of situational demands by asking participants to imagine how they would display emotions in each of the four situations. However, there are well-known limitations of this self-report approach, including self-presentation (such as social desirability), acquiescence and motivation, the capacity for individuals to predict their own behavior, and participant motivation. Reflecting broader issues with the over-reliance on self-report measurement in psychology (Monaghan & Bizumic, 2023; Rauthmann, 2023), this approach limits the capacity to differentiate the construct from its measurement. Therefore, multiple sources of data such as informant in addition to real-world observation, or even extensions using multi-trait multi-method and contemporary structural equation modeling variations should be considered, in addition to a larger array of convergent validity measures.

A strength of this study was that it used a representative sample of the UK population, going beyond the convenience sample of undergraduates that typifies psychological research. Online collection panels have been supported as a strong method of collecting representative data (Douglas et al., 2023; Litman et al., 2021); however, these panels are still likely to be biased toward individuals who are inclined and capable of regularly completing online surveys. Additionally, the present data are limited to one country. This was by design, to remove extraneous variables when establishing the model. However, now that we have a viable model, we can move forward to cross-cultural testing given differences in socio-cultural expressive norms. For example, comparing cultures that traditionally differ in overt emotional displays. Until this is established, generalization beyond the UK context should be considered carefully. Additionally, it’s important to note that the results hinge on peoples’ interpretation and understanding of the emotional terms used in the survey, which can vary based on factors like vocabulary depth and emotional intelligence. This variability might introduce another layer of complexity and potential bias to the findings. Future studies should aim to measure and control for these factors to refine the understanding of how individual differences in language and emotional perception influence display rules.

## Conclusions

The present study established a three-domain structure for expressive norms and generated a new scale, the ERS, to measure these domains. This structure highlights the importance of considering expressive norms as multidimensional. A key advantage of the ERS rating method is that it measures degrees of both suppression and amplification and provides a mid-point that allows raters to indicate when they would not modify an expression in either direction. These scores are easy to interpret: positive scores indicate the degree of amplification and negative scores indicate the degree of suppression. Overall, the work presented herein provides a strong foundation for the unifying understanding of expression regulation, with the capacity to bridge current gaps in understanding between the various fields invested in this topic.

## Supplemental Material

sj-docx-1-asm-10.1177_10731911251333664 – Supplemental material for The Expression Regulation Scale (ERS): Validation of Three Emotion Domains for Expressive Norms with Close and Distant Others in Private and Public Situations Supplemental material, sj-docx-1-asm-10.1177_10731911251333664 for The Expression Regulation Scale (ERS): Validation of Three Emotion Domains for Expressive Norms with Close and Distant Others in Private and Public Situations by Conal Monaghan, Yiyun Shou, Paige Mewton, Anika Quayle and Amy Dawel in Assessment

## Notes

1. To ensure a comprehensive analysis, we also tested a higher-order model in which vulnerable and disruptive emotions were hypothesized to load onto a higher-order factor. However, this model encountered several Heywood cases, indicated by negative latent variable variance estimates. Due to these estimation issues (resulting in impossible variance estimates), the higher-order model was deemed to be poorly specified.
