# Supplementary material for: The Expression Regulation Scale (ERS): Validation of Three Emotion Domains for Expressive Norms with Close and Distant Others in Private and Public Situations
Source: Assessment. 2025 May 6;33(3):439–57. doi: 10.1177/10731911251333664 (PMC12924882; doi:10.1177/10731911251333664)
Supplement: sj-docx-1-asm-10.1177_10731911251333664 – Supplemental material for The Expression Regulation Scale (ERS): Validation of Three Emotion Domains for Expressive Norms with Close and Distant Others in Private and Public Situations [file sj-docx-1-asm-10.1177_10731911251333664.docx]

**Supplementary Materials for:**

# The Expression Regulation Scale (ERS): Validation of three emotion domains for expressive norms with close and distant others in private and public situations

## Supplement A - Expression Regulation Scale (ERS)

This supplement presents the general format for the final 24-item ERS, scoring key, and R code for scoring the ERS subscales.

**Main task instructions:** The next few tasks ask how you think you should express emotions with people who are very close to you (for example, family and close friends) and people not so close to you (for example, colleagues and acquaintances) in private (for example, in either your own home or someone else's) and in public (for example, in a restaurant or park).

**Example specific instructions for private-close situation:**^[[1]](#footnote-2)^ For this task, we would like to know how you think you should^[[2]](#footnote-3)^ express emotions in **private** (for example, in either your own home or someone else's) with **people very close to you** (for example, family and close friends).

How should you express each of the following emotions in **private** with **people very close to you**?

Please make your rating anywhere on the scale from -100 to +100, where:

-100 = express no emotion/hide my emotion completely

-50 = express less than I feel

0 = express it as I feel it

+50 = express more than I feel

+100 = express much more than I feel

|  | Express no emotion/ hide my emotion completely | Express less than I feel | Express it as I feel it | Express more than I feel | Express much more than I feel |
| --- | --- | --- | --- | --- | --- |

|  | -100 | -50 | 0 | 50 | 100 |
| --- | --- | --- | --- | --- | --- |
| Admiration |  | | | | |
| Compassion |  | | | | |
| Delight |  | | | | |
| Excitement |  | | | | |
| Happiness |  | | | | |
| Hope |  | | | | |
| Pleasure |  | | | | |
| Pride |  | | | | |
| Despair |  | | | | |
| Distress |  | | | | |
| Embarrassment |  | | | | |
| Fear |  | | | | |
| Guilt |  | | | | |
| Hurt |  | | | | |
| Sadness |  | | | | |
| Unhappiness |  | | | | |
| Anger |  | | | | |
| Boredom |  | | | | |
| Disgust |  | | | | |
| Fury |  | | | | |
| Hatred |  | | | | |
| Irritation |  | | | | |
| Jealousy |  | | | | |
| Resentment |  | | | | |

**ERS scoring.** The ERS is scored by averaging the ratings within each subscale, allowing for up to one missing item per subscale.

Affiliative = mean(Admiration, Compassion, Delight, Excitement, Happiness, Hope, Pleasure, Pride)

Vulnerable = mean(Despair, Distress, Embarrassment, Fear, Guilt, Hurt, Sadness, Unhappiness

Disruptive = mean(Anger, Boredom, Disgust, Fury, Hatred, Irritation, Jealousy, Resentment)

The general R code for scoring the ERS is:

### Note: This code uses the dplyr package in R.

### Note: Variables are named in the standard format: prefix_Emotion, e.g., clspri_Admiration

### Note: Prefixes indicate the situation type as follows: clspri = close-private; clspub = close-public; dispri = distant-private; dispub = distant-public.

### Note. “Negative” is only used for researchers wishing to use the two-factor structure, which is the combined vulnerable and disruptive factors.

# Define lists of variables for each ERS subscale

aff_cols <- c("Admiration", "Compassion", "Delight", "Excitement", "Happiness", "Hope", "Pleasure", "Pride")

vul_cols <- c("Despair", "Distress", "Embarrassment", "Fear", "Guilt", "Hurt", "Sadness", "Unhappiness")

dis_cols <- c("Anger", "Boredom", "Disgust", "Fury", "Hatred", "Irritation", "Jealousy", "Resentment")

neg_cols <- c("Despair", "Distress", "Embarrassment", "Fear", "Guilt", "Hurt", "Sadness", "Unhappiness", "Anger", "Boredom", "Disgust", "Fury", "Hatred", "Irritation", "Jealousy", "Resentment")

# Function to calculate mean allowing for a maximum of one NA value

mean_oneNAmax <- function(x) {

if (sum(is.na(x)) > 1) {

return(NA)

} else {

return(mean(x, na.rm = TRUE))

}

}

# Calculate row means with the custom function

df <- df %>% # df is the dataframe

rowwise() %>%

mutate(

clspri_aff = mean_oneNAmax(c_across(matches(paste0("clspri_", aff_cols)))), # e.g., Calculates the affiliative subscale score for the close-private situation

clspri_vul = mean_oneNAmax(c_across(matches(paste0("clspri_", vul_cols)))),

clspri_dis = mean_oneNAmax(c_across(matches(paste0("clspri_", dis_cols)))),

clspri_neg = mean_oneNAmax(c_across(matches(paste0("clspri_", neg_cols)))),

clspub_aff = mean_oneNAmax(c_across(matches(paste0("clspub_", aff_cols)))),

clspub_vul = mean_oneNAmax(c_across(matches(paste0("clspub_", vul_cols)))),

clspub_dis = mean_oneNAmax(c_across(matches(paste0("clspub_", dis_cols)))),

clspub_neg = mean_oneNAmax(c_across(matches(paste0("clspub_", neg_cols)))),

dispri_aff = mean_oneNAmax(c_across(matches(paste0("dispri_", aff_cols)))),

dispri_vul = mean_oneNAmax(c_across(matches(paste0("dispri_", vul_cols)))),

dispri_dis = mean_oneNAmax(c_across(matches(paste0("dispri_", dis_cols)))),

dispri_neg = mean_oneNAmax(c_across(matches(paste0("dispri_", neg_cols)))),

dispub_aff = mean_oneNAmax(c_across(matches(paste0("dispub_", aff_cols)))),

dispub_vul = mean_oneNAmax(c_across(matches(paste0("dispub_", vul_cols)))),

dispub_dis = mean_oneNAmax(c_across(matches(paste0("dispub_", dis_cols)))),

dispub_neg = mean_oneNAmax(c_across(matches(paste0("dispub_", neg_cols))))

## Supplement B - ACO Heuristics and Final Emotions for Each Domain

### Table B1

#### ACO Heuristics and Final Emotions for Each Domain

| Domain | Emotion | Heuristic | 10-item selection | Final 8-item ERS |
| --- | --- | --- | --- | --- |
| Affiliative | |  |  |  |
|  | Admiration |  | Admiration | Admiration |
|  | Amusement |  |  |  |
|  | Awe |  |  |  |
|  | Calm |  |  |  |
|  | Compassion | X | Compassion | Compassion |
|  | Contentment |  |  |  |
|  | Curiosity |  |  |  |
|  | Delight |  | Delight | Delight |
|  | Desire |  |  |  |
|  | Elation |  |  |  |
|  | Enthusiasm |  |  |  |
|  | Excitement | X | Excitement | Excitement |
|  | Gratitude |  |  |  |
|  | Happiness | X | Happiness | Happiness |
|  | Hope | X | Hope | Hope |
|  | Interest |  |  |  |
|  | Joy |  |  |  |
|  | Love |  |  |  |
|  | Pleasure | X | Pleasure | Pleasure |
|  | Pride | X | Pride | Pride |
|  | Relaxation |  | Relaxation | Admiration |
|  | Relief | X |  |  |
|  | Surprise | X | Surprise |  |
|  | Sympathy | X |  |  |
|  | Triumph |  |  |  |
| Vulnerable | |  |  |  |
|  | Anxiety |  |  |  |
|  | Confusion |  |  |  |
|  | Depression |  |  |  |
|  | Despair | X | Despair | Despair |
|  | Disappointment | X | Disappointment |  |
|  | Distress | X | Distress | Distress |
|  | Embarrassment | X | Embarrassment | Embarrassment |
|  | Fear | X | Fear | Fear |
|  | Gloom |  |  |  |
|  | Grief |  |  |  |
|  | Guilt | X | Guilt | Guilt |
|  | Hopelessness |  |  |  |
|  | Hurt | X | Hurt | Hurt |
|  | Pain |  |  |  |
|  | Panic |  |  |  |
|  | Regret |  |  |  |
|  | Remorse |  |  |  |
|  | Sadness | X | Sadness | Sadness |
|  | Shame |  |  |  |
|  | Shock |  |  |  |
|  | Sorrow |  |  |  |
|  | Stress |  | Stress |  |
|  | Terror |  |  |  |
|  | Unhappiness |  | Unhappiness | Unhappiness |
|  | Worry |  |  |  |
|  | Pity |  |  |  |
| Disruptive | |  |  |  |
|  | Anger | X | Anger | Anger |
|  | Annoyance |  |  |  |
|  | Boredom |  | Boredom | Boredom |
|  | Contempt |  | Contempt |  |
|  | Disgust | X | Disgust | Disgust |
|  | Envy |  |  |  |
|  | Frustration | X | Frustration |  |
|  | Fury |  | Fury | Fury |
|  | Hatred |  | Hatred | Hatred |
|  | Irritation | X | Irritation | Irritation |
|  | Jealousy | X | Jealousy | Jealousy |
|  | Rage |  |  |  |
|  | Resentment |  | Resentment | Resentment |

*Note.* X indicates this emotion started with a heuristic of 15.

## Supplement C - Scree Plots from EFA

### Figure C

#### Scree Plots from EFA

*Note.* These figures were created using the Psych (Revelle, 2023) and GGplot2 (Wickham et al., 2016) package for R (R Core Team, 2021).

## Supplement D - EFA Results for 64 Emotions

### Table D

#### EFA Variance Explained and Inter-Factor Correlations for 64 Emotions

| Model |  |  |  | Proportion of variance explained | |  | Factor correlations | | |
| --- | --- | --- | --- | --- | --- | --- | --- | --- | --- |
| Structure | Situation | Factor |  | Total Variance | Explained Variance |  | 1 | 2 | 3 |
| Two-factor solution | | |  |  |  |  |  |  |  |
|  | Private-close | 1 |  | .33 | .56 |  | - | - | NA |
|  |  | 2 |  | .26 | .44 |  | .21 | - | NA |
|  | Private-distant | 1 |  | .32 | .63 |  | - | - | NA |
|  |  | 2 |  | .19 | .37 |  | .09 | - | NA |
|  | Public-close | 1 |  | .31 | .58 |  | - | - | NA |
|  |  | 2 |  | .22 | .42 |  | -.07 | - | NA |
|  | Public-distant | 1 |  | .32 | .63 |  | - | - | NA |
|  |  | 2 |  | .19 | .37 |  | .08 | - | NA |
|  |  | 1 |  |  |  |  |  |  |  |
| Three-factor solution | | |  |  |  |  |  |  |  |
|  | Private-close | 1 |  | .24 | .39 |  | - | - | - |
|  |  | 2 |  | .26 | .42 |  | .23 | - | - |
|  |  | 3 |  | .12 | .20 |  | .73 | .09 | - |
|  | Private-distant | 1 |  | .22 | .42 |  | - | - | - |
|  |  | 2 |  | .19 | .35 |  | .11 | - | - |
|  |  | 3 |  | .12 | .23 |  | .72 | .04 | - |
|  | Public-  close | 1 |  | .23 | .40 |  | - | - | - |
|  |  | 2 |  | .23 | .40 |  | -.04 | - | - |
|  |  | 3 |  | .11 | .20 |  | .68 | -.08 | - |
|  | Public-distant | 1 |  | .21 | .39 |  | - | - | - |
|  |  | 2 |  | .19 | .35 |  | .09 | - | - |
|  |  | 3 |  | .14 | .26 |  | .71 | .03 | - |

## Supplement E - Two-Factor Structure Analysis

Model fit estimates for the two-factor model were consistently lower than they were for the three-factor model, which was not surprising given the ACO identified optimum solutions for the three-factor, not the two-factor model. However, these results still suggest that the vulnerable and disruptive factors can reasonably be collapsed into a single factor where this appropriate to the researcher’s purpose and theoretical framework, particularly given the strong correlation between the disruptive and vulnerable factors.

For the two-factor model, the combined disruptive and vulnerable factor was named “Negative”, reflecting that these terms were all negatively valanced.

### Table E1

#### Comparison of Fit Estimates from Model Fit from Confirmatory Factor Analytic Models

|  | Model | Three-factor | | | | | | | | Two-factor | | | | | | |
| --- | --- | --- | --- | --- | --- | --- | --- | --- | --- | --- | --- | --- | --- | --- | --- | --- |
|  | Situation | χ^2^ | *df* | *p* | RMSEA | SRMR | CFI | NNFI |  | χ^2^ | *df* | *p* | RMSEA | SRMR | CFI | NNFI |
| Exploratory Sample | | | | | | | | | | | | | | | | |
|  | Private-close | 866.71 | 249 | <.001 | .058 | .053 | .956 | .952 |  | 1246.73 | 251 | <.001 | .079 | .063 | .917 | .909 |
|  | Private-distant | 590.15 | 249 | <.001 | .040 | .058 | .975 | .972 |  | 921.71 | 251 | <.001 | .064 | .063 | .933 | .927 |
|  | Public-close | 752.01 | 249 | <.001 | .054 | .046 | .959 | .954 |  | 1066.24 | 251 | <.001 | .073 | .052 | .924 | .916 |
|  | Public-distant | 732.63 | 249 | <.001 | .054 | .057 | .956 | .951 |  | 1124.5 | 251 | <.001 | .077 | .064 | .909 | .900 |
| Confirmatory Sample | | | | | | | | | | | | | | | | |
|  | Private-close | 841.77 | 249 | <.001 | .055 | .056 | .960 | .955 |  | 1218.04 | 251 | <.001 | .077 | .071 | .920 | .912 |
|  | Private-distant | 692.66 | 249 | <.001 | .049 | .054 | .960 | .956 |  | 949.23 | 251 | <.001 | .066 | .059 | .926 | .919 |
|  | Public-close | 732.21 | 249 | <.001 | .053 | .047 | .956 | .951 |  | 1046.49 | 251 | <.001 | .073 | .057 | .918 | .909 |
|  | Public-distant | 720.50 | 249 | <.001 | .052 | .047 | .955 | .951 |  | 955.74 | 251 | <.001 | .067 | .054 | .925 | .917 |

Note. Estimator = MLR. Both three and two factor results are presented for ease of comparison.

Estimates of measurement were marginally weaker for the two-factor model, ranging between .88 and .90, which was just below our .90 threshold (CFI estimates are interpreted as an indication of improvement over a null baseline model, rather than as a strict measure of good or bad fit using a specific threshold). This outcome was anticipated, given that the ACO had tailored the solution for a three-factor model. The ΔCFI for the three-factor model structure was comparable to that of the two-factor model, with the scalar invariance estimates falling between the more conservative and more widely used criteria.

For each sequential level of restriction (configural, metric, and scalar), the ERS was invariant across binary gender identities for all four situations, for both the three-factor (ΔCFI = .000 to .003) and two-factor (ΔCFI = .000 to .006) models.

### Table E2

#### Fit Estimates from Repeated Measures Invariance Modelling for Two and Three-Factor Emotion Domain Models

| Dataset |  | Three-factor | | | | |  | Two-factor | | | | |
| --- | --- | --- | --- | --- | --- | --- | --- | --- | --- | --- | --- | --- |
|  | Configural | χ^2^ | df | RMSEA | CFI | ΔCFI |  | χ^2^ | df | RMSEA | CFI | ΔCFI |
| Exploratory | | | | | | | | | | | | |
|  | Configural | 6544.54 | 4254 | .037 | .928 |  |  | 7514.005 | 4292 | .044 | .899 |  |
|  | Metric | 6627.36 | 4317 | .037 | .928 | .000 |  | 7594.806 | 4358 | .043 | .898 | .000 |
|  | Scalar | 6830.51 | 4380 | .038 | .938 | .004 |  | 7822.460 | 4424 | .044 | .884 | .003 |
| Confirmatory | | | | | | | | | | | | |
|  | Configural | 6704.74 | 4254 | .038 | .919 |  |  | 7520.418 | 4292 | .043 | .893 |  |
|  | Metric | 6798.34 | 4317 | .038 | .918 | .001 |  | 7624.013 | 4358 | .043 | .892 | .001 |
|  | Scalar | 7005.87 | 4380 | .039 | .913 | .005 |  | 7879.323 | 4424 | .044 | .886 | .006 |

*Note.* Estimator = MLR. Again, both factor structures are included for ease of comparison.

### Table E3

#### Mean Differences in Expressive Norms between Situations for the Combined Dataset

| Dimension | Affiliative | |  | Vulnerable | |  | Disruptive | |  | Negative | |
| --- | --- | --- | --- | --- | --- | --- | --- | --- | --- | --- | --- |
| Situation | Median (IQR) | M (SD) |  | Median (IQR) | M (SD) |  | Median (IQR) | M (SD) |  | Median (IQR) | M (SD) |
| Total | 10 (0, 31) | 17 (27) |  | -32 (-57, -10) | -34 (33) |  | -42 (-68, -18) | -42 (33) |  | -37 (-61, -15) | -38 (32) |
| Private-close | 14 (0, 39) | 24 (29) |  | -8 (-24, 0) | -10 (30) |  | -16 (-38, -1) | -20 (32) |  | -12 (-30, 0) | -15 (29) |
| Private-distant | 11 (0, 36) | 20 (26) |  | -30 (-52, -12) | -32 (29) |  | -41 (-65, -20) | -42 (30) |  | -36 (-56, -17) | -37 (28) |
| Public-close | 9 (0, 27) | 13 (24) |  | -43 (-66, -24) | -43 (29) |  | -53 (-75, -30) | -51 (30) |  | -48 (-69, -27) | -47 (28) |
| Public-distant | 7 (-2, 23) | 10 (26) |  | -49 (-72, -29) | -50 (28) |  | -58 (-81, -34) | -56 (30) |  | -53 (-75, -32) | -53 (28) |
| *p*-value | <0.001 | <0.001 |  | <0.001 | <0.001 |  | <0.001 | <0.001 |  | <0.001 | <0.001 |

*Note*. IQR = inter-quartile Range. Median differences were estimated using Kruskal-Wallis rank sum tests; mean differences were estimated using repeated measures ANOVA. See Supplementary Material F for breakdown of data into Exploratory and Confirmatory subsamples.

### Table E4

#### Associations Between ERS and the ERQ Suppression Subscale

|  | Situation | Affiliative | Vulnerable | Disruptive | Negative |
| --- | --- | --- | --- | --- | --- |
| ERQ suppression | | | | | |
|  | Private-close | -.06 | -.20*** | -.16*** | -.19*** |
|  | Private-distant | -.08* | -.19*** | -.13*** | -.17*** |
|  | Public-close | -.09** | -.12*** | -.05 | -.09** |
|  | Public-distant | -.08** | -.14*** | -.07* | -.11*** |

*Note.* * *p* < .05. ** *p* < .01. *** *p* < .001. Negative refers to the combined Vulnerable and Disruptive ERS dimensions. See Supplementary Material G for breakdown of data into Exploratory and Confirmatory subsamples.

## Supplement F - Parameter Estimates and Internal Consistencies for ERS Subscales

**Table F1**

*Parameter Estimates for the Private / Close Situation*

| **Dimension** | **Emotion** | **Estimate** |
| --- | --- | --- |
| Affiliative | Admiration | 0.801 |
| Affiliative | Compassion | 0.820 |
| Affiliative | Delight | 0.919 |
| Affiliative | Excitement | 0.901 |
| Affiliative | Happiness | 0.882 |
| Affiliative | Hope | 0.810 |
| Affiliative | Pleasure | 0.877 |
| Affiliative | Pride | 0.674 |
| Vulnerable | Despair | 0.808 |
| Vulnerable | Distress | 0.821 |
| Vulnerable | Embarrassment | 0.698 |
| Vulnerable | Fear | 0.813 |
| Vulnerable | Guilt | 0.697 |
| Vulnerable | Hurt | 0.777 |
| Vulnerable | Sadness | 0.730 |
| Vulnerable | Unhappiness | 0.779 |
| Disruptive | Anger | 0.805 |
| Disruptive | Boredom | 0.643 |
| Disruptive | Disgust | 0.771 |
| Disruptive | Fury | 0.797 |
| Disruptive | Hatred | 0.852 |
| Disruptive | Irritation | 0.766 |
| Disruptive | Jealousy | 0.742 |
| Disruptive | Resentment | 0.816 |

*Note.* Parameter estimates are standardized. Estimates are from the Confirmatory Sample only

**Table F2**

*Parameter Estimates for the Private / Distant Situation*

| **Dimension** | **Emotion** | **Estimate** |
| --- | --- | --- |
| Affiliative | Admiration | 0.809 |
| Affiliative | Compassion | 0.721 |
| Affiliative | Delight | 0.839 |
| Affiliative | Excitement | 0.833 |
| Affiliative | Happiness | 0.841 |
| Affiliative | Hope | 0.683 |
| Affiliative | Pleasure | 0.755 |
| Affiliative | Pride | 0.467 |
| Vulnerable | Despair | 0.812 |
| Vulnerable | Distress | 0.799 |
| Vulnerable | Embarrassment | 0.652 |
| Vulnerable | Fear | 0.739 |
| Vulnerable | Guilt | 0.709 |
| Vulnerable | Hurt | 0.772 |
| Vulnerable | Sadness | 0.704 |
| Vulnerable | Unhappiness | 0.802 |
| Disruptive | Anger | 0.793 |
| Disruptive | Boredom | 0.642 |
| Disruptive | Disgust | 0.757 |
| Disruptive | Fury | 0.840 |
| Disruptive | Hatred | 0.789 |
| Disruptive | Irritation | 0.738 |
| Disruptive | Jealousy | 0.740 |
| Disruptive | Resentment | 0.786 |

*Note.* Parameter estimates are standardized. Estimates are from the Confirmatory Sample only

**Table F3**

*Parameter Estimates for the Public / Close Situation*

| **Dimension** |  | **Emotion** | **Estimate** |
| --- | --- | --- | --- |
| Affiliative |  | Admiration | 0.808 |
| Affiliative |  | Compassion | 0.743 |
| Affiliative |  | Delight | 0.854 |
| Affiliative |  | Excitement | 0.832 |
| Affiliative |  | Happiness | 0.852 |
| Affiliative |  | Hope | 0.786 |
| Affiliative |  | Pleasure | 0.785 |
| Affiliative |  | Pride | 0.588 |
| Vulnerable |  | Despair | 0.800 |
| Vulnerable |  | Distress | 0.797 |
| Vulnerable |  | Embarrassment | 0.643 |
| Vulnerable |  | Fear | 0.757 |
| Vulnerable |  | Guilt | 0.694 |
| Vulnerable |  | Hurt | 0.826 |
| Vulnerable |  | Sadness | 0.724 |
| Vulnerable |  | Unhappiness | 0.823 |
| Disruptive |  | Anger | 0.827 |
| Disruptive |  | Boredom | 0.621 |
| Disruptive |  | Disgust | 0.751 |
| Disruptive |  | Fury | 0.765 |
| Disruptive |  | Hatred | 0.815 |
| Disruptive |  | Irritation | 0.781 |
| Disruptive |  | Jealousy | 0.760 |
| Disruptive |  | Resentment | 0.807 |

*Note.* Parameter estimates are standardized. Estimates are from the Confirmatory Sample only

**Table F4**

*Parameter Estimates for the Public / Distant Situation*

| **Dimension** | **Emotion** | **Estimate** |
| --- | --- | --- |
| Affiliative | Admiration | 0.781 |
| Affiliative | Compassion | 0.722 |
| Affiliative | Delight | 0.845 |
| Affiliative | Excitement | 0.851 |
| Affiliative | Happiness | 0.827 |
| Affiliative | Hope | 0.762 |
| Affiliative | Pleasure | 0.782 |
| Affiliative | Pride | 0.558 |
| Vulnerable | Despair | 0.801 |
| Vulnerable | Distress | 0.784 |
| Vulnerable | Embarrassment | 0.626 |
| Vulnerable | Fear | 0.743 |
| Vulnerable | Guilt | 0.687 |
| Vulnerable | Hurt | 0.720 |
| Vulnerable | Sadness | 0.743 |
| Vulnerable | Unhappiness | 0.756 |
| Disruptive | Anger | 0.824 |
| Disruptive | Boredom | 0.610 |
| Disruptive | Disgust | 0.798 |
| Disruptive | Fury | 0.808 |
| Disruptive | Hatred | 0.787 |
| Disruptive | Irritation | 0.749 |
| Disruptive | Jealousy | 0.721 |
| Disruptive | Resentment | 0.764 |

*Note.* Parameter estimates are standardized. Estimates are from the Confirmatory Sample only

### Table F5

#### Internal Consistencies for ERS Subscales in Exploratory Sample

| Situation | | Subscale | | |  | Correlations | | |
| --- | --- | --- | --- | --- | --- | --- | --- | --- |
|  | Estimate | Affiliative | Disruptive | Vulnerable |  | Subscale | Disruptive | Vulnerable |
| Private-close | | | | | | | | |
|  | Alpha | .95 | .92 | .93 |  | Affiliative | -.04 | .01 |
|  | Omega | .95 | .92 | .93 |  | Disruptive | - | .67 |
| Private-distant | | | | | | | | |
|  | Alpha | .91 | .93 | .92 |  | Affiliative | .00 | -.03 |
|  | Omega | .91 | .93 | .92 |  | Disruptive | - | .78 |
| Public-close | | | | | | | | |
|  | Alpha | .94 | .93 | .92 |  | Affiliative | -.14 | -.16 |
|  | Omega | .94 | .93 | .92 |  | Disruptive | - | .76 |
| Public-distant | | | | | | | | |
|  | Alpha | .91 | .93 | .92 |  | Affiliative | -.03 | -.01 |
|  | Omega | .91 | .94 | .92 |  | Disruptive | - | .79 |

*Note.* Internal consistencies for the combined vulnerable and Disruptive (negative valence) factor were between .95 and .96. Omega refers to total Omega, given the one-factor structure.

### Table F6

#### Internal Consistencies for ERS Subscales in Confirmatory Sample

| Situation | | Subscale | | |  | Correlations | | |
| --- | --- | --- | --- | --- | --- | --- | --- | --- |
|  | Estimate | Affiliative | Disruptive | Vulnerable |  | Subscale | Disruptive | Vulnerable |
| Private-close | | | | | | | | |
|  | Alpha | .95 | .92 | .92 |  | Affiliative | -.07 | .03 |
|  | Omega | .95 | .92 | .92 |  | Disruptive | - | .69 |
| Private-distant | | | | | | | | |
|  | Alpha | .91 | .92 | .91 |  | Affiliative | -.06 | -.05 |
|  | Omega | .91 | .92 | .91 |  | Disruptive | - | .75 |
| Public-close | | | | | | | | |
|  | Alpha | .93 | .92 | .92 |  | Affiliative | -.05 | -.02 |
|  | Omega | .93 | .92 | .92 |  | Disruptive | - | .75 |
| Public-distant | | | | | | | | |
|  | Alpha | .92 | .91 | .90 |  | Affiliative | .02 | .10 |
|  | Omega | .92 | .92 | .90 |  | Disruptive | - | .78 |

*Note.* Internal consistencies for the combined vulnerable and Disruptive (negative valence) factor were between .95 for all situations.

## Supplement G - Age and Gender Identity Invariance Model Output

### Table G1

*Multigroup Confirmatory Factor Analysis Output for Binary Gender Identity*

|  |  | Three-Factor Data | | | | |  | Two-Factor Data | | | | |  |
| --- | --- | --- | --- | --- | --- | --- | --- | --- | --- | --- | --- | --- | --- |
| Situation | Model | χ^2^ | df | RMSEA | CFI | ΔCFI |  | Model | χ^2^ | df | RMSEA | CFI | ΔCFI |
| Close Private | Configural | 959.625 | 498 | .058 | .955 |  |  | Configural | 1374.254 | 502 | .080 | .915 |  |
|  | Metric | 989.943 | 519 | .057 | .955 | .000 |  | Metric | 1411.777 | 524 | .078 | .915 | .000 |
|  | Scalar | 1025.561 | 540 | .056 | .954 | -.001 |  | Scalar | 1465.947 | 546 | .077 | .913 | -.001 |
| Distant Private | Configural | 873.071 | 498 | .049 | .961 |  |  | Configural | 1221.666 | 502 | .067 | .925 |  |
|  | Metric | 909.228 | 519 | .048 | .960 | -.001 |  | Metric | 1258.971 | 524 | .066 | .925 | -.001 |
|  | Scalar | 958.819 | 540 | .049 | .958 | -.002 |  | Scalar | 1351.138 | 546 | .067 | .919 | -.006 |
| Close Public | Configural | 904.625 | 498 | .050 | .963 |  |  | Configural | 1312.166 | 502 | .070 | .926 |  |
|  | Metric | 945.960 | 519 | .050 | .962 | -.001 |  | Metric | 1354.371 | 524 | .069 | .925 | -.001 |
|  | Scalar | 997.684 | 540 | .050 | .959 | -.003 |  | Scalar | 1440.029 | 546 | .070 | .920 | -.005 |
| Distant Public | Configural | 992.546 | 498 | .055 | .951 | .000 |  | Configural | 429.157 | 206 | .059 | .964 |  |
|  | Metric | 1025.831 | 519 | .054 | .951 | -.001 |  | Metric | 453.062 | 220 | .058 | .963 | -.001 |
|  | Scalar | 1076.465 | 540 | .054 | .948 | -.002 |  | Scalar | 484.581 | 234 | .057 | .961 | -.002 |

*Note.* Estimator = MLR. All chi^2^ were significant and *p* < .001.

### Table G2

*Multigroup Confirmatory Factor Analysis Output for Age Groups*

|  |  | Three-Factor Data | | | | | |  | | Two-Factor Data | | | | | |  | |
| --- | --- | --- | --- | --- | --- | --- | --- | --- | --- | --- | --- | --- | --- | --- | --- | --- | --- |
| Situation | Model | χ^2^ | df | RMSEA | CFI | ΔCFI |  | | Model | | χ^2^ | df | RMSEA | CFI | ΔCFI | |  |
| Close Private | Configural | 1362.086 | 747 | .064 | .946 |  |  | | Configural | | 1806.025 | 753 | .084 | .908 |  | |  |
|  | Metric | 1413.422 | 789 | .062 | .946 | .000 |  | | Metric | | 1865.429 | 797 | .081 | .907 | .000 | |  |
|  | Scalar | 1526.362 | 831 | .063 | .941 | -.005 |  | | Scalar | | 1987.325 | 841 | .081 | .903 | -.005 | |  |
| Distant Private | Configural | 1219.579 | 747 | .052 | .957 |  |  | | Configural | | 1612.542 | 753 | .070 | .921 |  | |  |
|  | Metric | 1263.730 | 789 | .050 | .958 | .001 |  | | Metric | | 1670.844 | 797 | .068 | .921 | .000 | |  |
|  | Scalar | 1401.380 | 831 | .053 | .950 | -.008 |  | | Scalar | | 1819.169 | 841 | .070 | .913 | -.008 | |  |
| Close Public | Configural | 1318.937 | 747 | .057 | .954 |  |  | | Configural | | 1763.489 | 753 | .075 | .918 |  | |  |
|  | Metric | 1397.572 | 789 | .056 | .951 | -.003 |  | | Metric | | 1841.815 | 797 | .074 | .916 | -.002 | |  |
|  | Scalar | 1533.584 | 831 | .059 | .944 | -.007 |  | | Scalar | | 1991.123 | 841 | .075 | .909 | -.007 | |  |
| Distant Public | Configural | 1251.781 | 747 | .054 | .955 |  |  | | Configural | | 511.645 | 309 | .054 | .970 |  | |  |
|  | Metric | 1316.764 | 789 | .053 | .953 | -.002 |  | | Metric | | 556.217 | 337 | .054 | .968 | -.002 | |  |
|  | Scalar | 1460.331 | 831 | .056 | .945 | -.008 |  | | Scalar | | 630.807 | 365 | .056 | .962 | -.006 | |  |

*Note.* Estimator = MLR. All c^2^ were significant and *p* < .001. Age categories are < -1 SD, -1 to 1 SD, and > 1 SD.

## Supplement H - Estimates of Central Tendency and Dispersion for ERS Subscales

### Table H1

#### Estimates of Central Tendency and Dispersion for ERS Subscales in Exploratory Sample

| ERS subscale | | Total | Close-  private | Close-  public | Distant-  private | Distant-  public | *p*-value |
| --- | --- | --- | --- | --- | --- | --- | --- |
| Affiliative | | | | | | | |
|  | Median (IQR) | 10 (0, 31) | 16 (0, 40) | 12 (0, 37) | 9 (0, 27) | 7 (0, 21) | <0.001 |
|  | Mean (SD) | 17 (27) | 25 (29) | 20 (27) | 13 (24) | 9 (26) | <0.001 |
| Vulnerable | | | | | | | |
|  | Median (IQR) | -32 (-57, -11) | -8 (-24, 0) | -32 (-52, -13) | -41 (-67, -24) | -47 (-73, -28) | <0.001 |
|  | Mean (SD) | -34 (32) | -11 (30) | -34 (28) | -43 (29) | -49 (29) | <0.001 |
| Disruptive | | | | | | | |
|  | Median (IQR) | -41 (-69, -17) | -15 (-37, 0) | -41 (-67, -21) | -53 (-76, -30) | -58 (-80, -33) | <0.001 |
|  | Mean (SD) | -42 (33) | -20 (31) | -43 (30) | -51 (30) | -55 (30) | <0.001 |
| Negative | | | | | | | |
|  | Median (IQR) | -37 (-61, -15) | -12 (-29, 0) | -37 (-57, -19) | -45 (-70, -27) | -52 (-76, -30) | <0.001 |
|  | Mean (SD) | -38 (31) | -15 (29) | -38 (28) | -47 (28) | -52 (28) | <0.001 |

*Note.* IQR = inter-quartile Range. Medians differences indicated with Kruskal-Wallis rank sum test, mean differences estimated using repeated-measures ANOVA.

### Table H2

#### Estimates of Central Tendency and Dispersion for ERS Subscales in Confirmatory Sample

| ERS subscale | | Total | Close-  private | Close-  public | Distant-  private | Distant-  public | *p*-value |
| --- | --- | --- | --- | --- | --- | --- | --- |
| Affiliative | | | | | | | |
|  | Median (IQR) | 9 (0, 30) | 13 (1, 39) | 11 (0, 35) | 8 (0, 27) | 6 (-4, 25) | <.001 |
|  | Mean (SD) | 17 (27) | 24 (29) | 19 (26) | 14 (24) | 10 (26) | <.001 |
| Vulnerable | | | | | | | |
|  | Median (IQR) | -31 (-58, -10) | -8 (-24, 0) | -27 (-51, -11) | -44 (-66, -24) | -50 (-71, -30) | <.001 |
|  | Mean (SD) | -34 (33) | -10 (29) | -31 (29) | -44 (29) | -50 (28) | <.001 |
| Disruptive | | | | | | | |
|  | Median (IQR) | -43 (-66, -18) | -17 (-39, -2) | -40 (-62, -19) | -53 (-74, -29) | -58 (-81, -36) | <.001 |
|  | Mean (SD) | -42 (33) | -20 (32) | -40 (30) | -51 (30) | -56 (29) | <.001 |
| Negative | | | | | | | |
|  | Median (IQR) | -37 (-61, -16) | -13 (-32, 0) | -35 (-55, -16) | -49 (-68, -27) | -54 (-75, -33) | <.001 |
|  | Mean (SD) | -38 (32) | -15 (29) | -36 (28) | -47 (28) | -53 (27) | <.001 |

*Note.* IQR = inter-quartile Range. Medians differences indicated with Kruskal-Wallis rank sum test, mean differences estimated using repeated-measures ANOVA.

## Supplement I - Latent ANOVA Results

## Table I1

## *Significance Tests for Main and Interaction Effects Using L-RM-ANOVA (Affiliative Domain)*

| Term | Test | Test Statistic | *p* - value |
| --- | --- | --- | --- |
| Main Effect Intercept | *F*-test | 647.03 | <.001 |
|  | Wald | 628.51 |  |
|  | *approx. F* | 628.51 | <.001 |
| Main Effect Interactant | *F*-test | 225.48 | <.001 |
|  | Wald | 205.91 |  |
|  | *approx. F* | 205.91 | <.001 |
| Main Effect Context | *F*-test |  | <.001 |
|  | Wald | 74.131 |  |
|  | *approx. F* | 74.131 | <.001 |
| Interaction | *F*-test | 1.36 | .244 |
|  | Wald | 1.06 |  |
|  | *approx. F* | 1.36 | .30 |

*Note.* df1 = 1 and df2 = 1-12 for all models. F and Wald are derived from the Structural Equation Modelling Framework.

## Table I2

## *Significance Tests for Main and Interaction Effects Using L-RM-ANOVA (Vulnerable Domain)*

| Term | Test | Test Statistic | *p* - value |
| --- | --- | --- | --- |
| Main Effect Intercept | *F*-test | 2301.40 | <.001 |
|  | Wald | 2220.84 |  |
|  | *approx. F* | 2220.80 | <.001 |
| Main Effect Interactant | *F*-test | 1057.20 | <.001 |
|  | Wald | 948.80 |  |
|  | *approx. F* | 948.80 | <.001 |
| Main Effect Context | *F*-test | 832.25 | <.001 |
|  | Wald | 651.61 |  |
|  | *approx. F* | 651.61 | <.001 |
| Interaction | *F*-test | 285.63 | <.001 |
|  | Wald | 220.05 |  |
|  | *approx. F* | 220.05 | <.001 |

*Note.* df1 = 1 and df2 = 1-12 for all models. F and Wald are derived from the Structural Equation Modelling Framework.

## Table I3

## *Significance Tests for Main and Interaction Effects Using L-RM-ANOVA (Disruptive Domain)*

| Term | Test | Test Statistic | *p* - value |
| --- | --- | --- | --- |
| Main Effect Intercept | *F*-test | 3252.40 | <.001 |
|  | Wald | 3141.53 |  |
|  | *approx. F* | 3141.50 | <.001 |
| Main Effect Interactant | *F*-test | 801.81 | <.001 |
|  | Wald | 722.38 |  |
|  | *approx. F* | 722.38 | <.001 |
| Main Effect Context | *F*-test | 652.70 | <.001 |
|  | Wald | 525.20 |  |
|  | *approx. F* | 525.20 | <.001 |
| Interaction | *F*-test | 337.97 | <.001 |
|  | Wald | 262.32 |  |
|  | *approx. F* | 262.32 | <.001 |

*Note.* df1 = 1 and df2 = 1-12 for all models. F and Wald are derived from the Structural Equation Modelling Framework.

## Supplement J - Associations with External Variables

### Table J1

#### Associations with External Variables in Exploratory Sample

| External variable | | ERS subscale | | | |
| --- | --- | --- | --- | --- | --- |
|  | Situation | Affiliative | Disruptive | Vulnerable | Negative |
| ERQ-suppression | | | | | |
|  | Private-close | .079 | -.084 | -.050 | -.069 |
|  | Private-distant | .120** | -.108* | -.102* | -.109* |
|  | Public-close | .062 | -.051 | -.037 | -.046 |
|  | Public-distant | .123** | -.056 | -.042 | -.052 |
| ERQ-reappraisal | | | | | |
|  | Private-close | -.046 | -.105* | -.196*** | -.156*** |
|  | Private-distant | -.060 | -.078 | -.170*** | -.130** |
|  | Public-close | -.072 | -.006 | -.095* | -.052 |
|  | Public-distant | -.080 | -.055 | -.138** | -.101* |

*Note.* * *p* < .05. ** *p* < .01. *** *p* < .001.

**Table J2**

#### Associations with External Variables in Confirmatory Sample

| External variable | | ERS subscale | | | |
| --- | --- | --- | --- | --- | --- |
|  | Situation | Affiliative | Disruptive | Vulnerable | Negative |
| ERQ-suppression | | | | | |
|  | Private-close | .080 | -.103* | -.026 | -.070 |
|  | Private-distant | .101* | -.143** | -.092* | -.124** |
|  | Public-close | .112* | -.056 | -.061 | -.062 |
|  | Public-distant | .102* | -.063 | -.022 | -.045 |
| ERQ-reappraisal | | | | | |
|  | Private-close | -.076 | -.211*** | -.195*** | -.216*** |
|  | Private-distant | -.092* | -.172*** | -.212*** | -.203*** |
|  | Public-close | -.102* | -.092* | -.142** | -.123** |
|  | Public-distant | -.081 | -.092* | -.136** | -.120** |

*Note.* * *p* < .05. ** *p* < .01. *** *p* < .001.

1. The general format for the specific instructions for each situation is: For this task, we would like to know how you think you should express emotions in <context> (for example, <define context>) with <interactant> (for example, <define interactant>). Please take a moment, while the below clock counts down, to imagine interacting in <context> with <interactant>. How should you express each of the following emotions in <context> with <interactant>? [↑](#footnote-ref-2)
2. The ERS has been validated for people’s beliefs about how they should express emotions, to capture display rule norms. However, the ERS wording could be adapted to capture expressive behaviour by replacing “how you think you should express emotions” and “How should you express each…” with “how you express emotions” and “How do you express each…” respectively. The wording could also be adapted to capture habitual expression regulation, akin to that captured by the Emotion Regulation Questionnaire (ERQ; Gross & John 2003); for example, by asking “When I feel the following emotions, I…”. However, the factor structure of the ERS is yet to be tested for these alternative wordings and thus there will be a need to reassess the validity of adapted versions of the scales. [↑](#footnote-ref-3)
